# Gastrointestinal Microbiota Imbalance and Sensory Processing Dysregulation in Autism Spectrum Disorder: A Systematic Review

**DOI:** 10.7759/cureus.114142

**Published:** 2026-08-07

**Authors:** Felicia T Bonner-Reid

**Affiliations:** 1 Medicine and Surgery, University of Medical Sciences of Granma (Celia Sánchez Manduley), Manzanillo, CUB

**Keywords:** autism spectrum disorder, gastrointestinal microbiota, microbial dysbiosis, neurodevelopmental disorder, sensory dysregulation

## Abstract

Autism spectrum disorder (ASD) is a neurodevelopmental disorder characterized by challenges with social communication and repetitive behaviors. Many people with ASD also experience gastrointestinal symptoms and sensory processing dysfunction. New research has identified a potential role for microbial dysbiosis (imbalance of gut microbiota) in these comorbidities. This systematic review aimed to explore the links between gut microbiota composition, gastrointestinal symptoms, sensory processing differences, and dietary patterns in individuals with ASD, as well as the potential for a bidirectional relationship and dietary-based interventions targeting the microbiota. PubMed, Scopus, Web of Science, and Google Scholar were used to retrieve publications from 2011 to 2026, and a systematic review was conducted. Studies were chosen that examined gut microbiota, gastrointestinal symptoms, and sensory processing in people with ASD. Only intervention and observational studies were included. Many people with ASD reported gastrointestinal symptoms, sensory processing differences, restricted dietary patterns, and gut microbiota composition. However, the majority of studies included were cross-sectional, which does not allow for an inference of temporal direction or causality. The evidence indicates there may be a bidirectional relationship between gastrointestinal and sensory symptoms and microbial differences, and the sensory symptoms, food selectivity, food restriction, stool consistency, use of medication, and clinical characteristics may also affect the composition of the microbiota. Initial microbiome interventions had some potential for improvement in selected outcomes, but with limited confidence due to small sample sizes, heterogeneity in methods used, and potential for bias. The current evidence suggests that changes in the gut microbiota composition are associated with gastrointestinal symptoms, sensory dysregulation, and dietary patterns in ASD, but does not provide evidence to indicate that microbial dysbiosis is a primary mechanism. The possibility of reverse causation exists, as the food environment could be influenced by sensory sensitivity and/or eating habits, and, in turn, these factors could affect the composition of the microbiota. To better understand directionality and the efficacy of microbiome-targeted interventions, longitudinal studies and sufficiently powered randomized controlled trials are warranted.

## Introduction and background

Autism spectrum disorder (ASD) is a complex neurodevelopmental disorder characterized by challenges in social communication, interests, and repetitive behaviors [1]. It is ranked among the top causes of nonfatal health burden for affected individuals, with the lifetime cost in the United States estimated at $4 trillion and projected to increase to $15 trillion by 2029 [2]. The rising prevalence of ASD continues to contribute to a significant financial burden, highlighting the critical importance of early diagnosis and intervention to mitigate long-term societal and individual impacts [2]. ASD is ranked in the top 10 causes of nonfatal health burden, younger than 20 years, with an estimated 61.8 million individuals. The Global Burden of Disease estimates that about one out of 127 people in the world was diagnosed with ASD in 2021. This is an average worldwide number, and prevalence varies widely between studies, with it being unclear in many low- and middle-income countries [3].

Prompt diagnosis and intervention can enhance the developmental and functional outcomes for persons with ASD; however, timely diagnosis continues to be a challenge. The Centers for Disease Control and Prevention (CDC) Autism and Developmental Disabilities Monitoring (ADDM) Network estimates that in 2022, 16 surveillance sites across the United States identified one out of every 31 (3.2%) children aged eight years with ASD. This estimate is based on surveillance data from the communities that participated in the US surveillance system and should not be viewed as a global prevalence estimate [4,5]. ASD is usually diagnosed by behavioral observations and by using standardized assessment instruments like the Autism Diagnostic Observation Schedule (ADOS-2) and the Autism Diagnostic Interview-Revised (ADI-R). It is categorized as having different severity levels (levels 1-3: mild to severe) of social communication deficits and restricted/repetitive behaviors as per the criteria laid down by the Diagnostic and Statistical Manual of Mental Disorders, Fifth Edition (DSM-5) [6]. ASD is often accompanied by gastrointestinal symptoms, which include constipation, diarrhea, abdominal pain, and bloating. Results of prevalence estimates, however, depend greatly on the type of participants, type of sample, clinical population, assessment method, and definition of gastrointestinal symptoms and can range from as low as 0% to as high as 90% and appear to be high in selected clinical populations [7]. Dysfunctions in the microbiota-gut-brain axis (communication between the gut microbiota, immune system, and central nervous system) have been linked to these gastrointestinal disorders [8].

The gut-brain axis is a critical pathway that affects the physiology and neuropsychology of human beings and has recently been associated with changes in sensory processing in people with ASD [9]. The presence of microbial dysbiosis in people with ASD has been linked to behavioral traits such as sensory sensitivities, but the directionality and causal relationship of this association are not clear [10]. It has been suggested that the gut-brain axis could be a mechanism linking microbial dysbiosis and sensory processing differences in individuals with ASD. Sensory dysregulation in ASD can be described as hypersensitivity (over-responsiveness), hyposensitivity (under-responsiveness), and sensory seeking behaviors. Hypersensitivity can be expressed as avoidance of loud noises, hyposensitivity as reduced response to pain or temperature, and sensory seeking behavior as repetitive touching or movement to help regulate sensory input [11].

Hyperreactive or hyporeactive response to sensory stimuli, including sound, light, and tactile input, is a frequent comorbid condition in children and adults with ASD [12]. But there are inconsistencies in the available literature when it comes to prevalence/incidence for sensory dysregulation as it pertains to ASD. The clinical manifestations of sensory dysregulation in individuals with ASD highlight both hypersensitivity and hyposensitivity to sensory stimuli like light, sound, and touch.

Several interventions have been shown to mitigate sensory dysregulation in individuals with ASD. Improvements in sensory processing, adaptive behaviors, and daily functioning have been shown with occupational therapy, feeding therapy, and aquatic therapy. Occupational therapy with sensory integration, for example, can help decrease tactile and auditory oversensitivity, and aquatic therapy can help to enhance behavioral outcomes and proprioceptive regulation [13].

The relationship between gut health and sensory processing has also been of great interest over the last few years, as gastrointestinal dysfunction is increasingly recognized in people with ASD [14]. For example, children with ASD are food selective, have abnormal eating patterns, and may experience heightened gastrointestinal discomfort [14,15]. This connection indicates that dysbiosis of the gut flora could affect gastrointestinal function and may also play a part in sensory processing dysregulation [16]. Moreover, some recent research suggests that compromised immune system changes, such as changes in cytokine activity in the gut and brain, have also been described in connection with sensory processing problems with ASD [17].

This systematic review aims (1) to investigate the association between gut microbiota disturbances and sensory processing dysfunction in individuals with ASD, considering the multitude of factors that contribute to the etiology of ASD and the microbiota-gut-brain axis which has recently attracted significant interest in the field, (2) to address the literature gaps, (3) to provide insights into the therapeutic potential of the gut-brain axis in alleviating symptoms in ASD patients, and (4) to enhance the understanding of the relationship between gut health and sensory processing.

## Review

Methodology

Study Design

This study was designed as a systematic review to explore the connection between gastrointestinal microbiota imbalance and sensory processing dysregulation among people with ASD. The review process was conducted following the Preferred Reporting Items for Systematic Reviews and Meta-Analyses (PRISMA) guidelines to ensure clarity in the research process and to make it systematic [18]. The purpose of this study was to gather and synthesize all available evidence on changes in gut microbiota, gastrointestinal symptoms, sensory processing difficulties, and microbiota-based interventions in people with ASD. Observational studies and intervention studies were both included to gain a better understanding of the relationship between the gut microbiota and sensory processing in ASD.

PICOS (Population, Intervention/Exposure, Comparator, Outcomes, and Study Design) Framework

The PICOS framework was applied to the development of the review question, search strategy, eligibility criteria, and study selection process. This population consisted of children, adolescents, and young adults diagnosed with ASD. The intervention/exposure comprised gastrointestinal microbiota imbalance, microbial dysbiosis, gastrointestinal symptoms, dietary patterns, food selectivity, and interventions targeting the microbiome including probiotics, synbiotics, and fecal microbiota transplantation. Comparator groups consisted of typically developing controls, healthy controls, ASD with no severe gastrointestinal symptoms, placebo groups, baseline or pre-intervention status, and standard care groups when applicable. The main outcomes of interest were sensory processing dysregulation, sensory over-responsivity or hyporesponsivity, gastrointestinal symptoms, changes in gut microbiota composition, microbial diversity, metabolomic alterations, anxiety, behavioral symptoms, and ASD-related symptom severity. The study designs considered were observational studies, cross-sectional studies, longitudinal studies, randomized controlled trials, and non-randomized intervention studies published between 2011 and 2026.

Literature Search and Search Strategy

A thorough electronic literature search was carried out in PubMed/MEDLINE, Scopus, Web of Science Core Collection, and Embase. Google Scholar was used as a secondary source to find potentially relevant studies that might not have been included in the main bibliographic databases. The last search took place on 28 May 2026.

Two reviewers independently developed the search strategy to meet the objectives of the review. Controlled vocabulary, such as Medical Subject Headings in PubMed/MEDLINE and Emtree in Embase, was used in conjunction with free-text keywords. The main search terms were "autism spectrum disorder", "gut microbiota", "microbial dysbiosis", "sensory processing dysfunction", "gastrointestinal symptoms", and "gut-brain axis". Information was combined in the following ways: within each concept, the synonyms were connected by the word "OR" and the core concepts were connected by the word "AND".

Search syntax was modified for each database to reflect the indexing system and field codes used in that database. All controlled vocabulary, free-text, Boolean, field tag, filters, and the number of records found in each database are included in the complete database-specific search strategies (Table 1).

**Table 1:** Complete database-specific electronic search strategies ASD: autism spectrum disorder; MeSH: Medical Subject Headings; TS: Topic Search; TITLE-ABS-KEY: title, abstract, and keywords; ti: title; ab: abstract; kw: keywords

Database/source	Complete search strategy	Limits and filters applied	Screening depth/records retrieved
PubMed/MEDLINE	("Autism Spectrum Disorder"[MeSH Terms] OR "autism spectrum disorder"[Title/Abstract] OR ASD[Title/Abstract]) AND ("Gastrointestinal Microbiome"[MeSH Terms] OR "gut microbiota"[Title/Abstract] OR "intestinal microbiome"[Title/Abstract] OR "microbial dysbiosis"[Title/Abstract]) AND ("sensory processing"[Title/Abstract] OR "sensory processing dysfunction"[Title/Abstract] OR "sensory dysregulation"[Title/Abstract] OR "sensory sensitivity"[Title/Abstract]) AND ("Gastrointestinal Diseases"[MeSH Terms] OR "gastrointestinal symptoms"[Title/Abstract]) AND ("gut-brain axis"[Title/Abstract] OR "microbiota-gut-brain axis"[Title/Abstract])	English language; publication period from 1 January 2011 to 28 May 2026; no geographical restriction	Records retrieved: n=420
Scopus	TITLE-ABS-KEY("autism spectrum disorder" OR ASD) AND TITLE-ABS-KEY("gut microbiota" OR "intestinal microbiome" OR "microbial dysbiosis") AND TITLE-ABS-KEY("sensory processing" OR "sensory processing dysfunction" OR "sensory dysregulation" OR "sensory sensitivity") AND TITLE-ABS-KEY("gastrointestinal diseases" OR "gastrointestinal symptoms") AND TITLE-ABS-KEY("gut-brain axis" OR "microbiota-gut-brain axis") AND PUBYEAR > 2010 AND PUBYEAR < 2027 AND LANGUAGE(English)	English language; publication years 2011-2026; no geographical restriction	Records retrieved: n=310
Web of Science Core Collection	TS=("autism spectrum disorder" OR ASD) AND TS=("gut microbiota" OR "intestinal microbiome" OR "microbial dysbiosis") AND TS=("sensory processing" OR "sensory processing dysfunction" OR "sensory dysregulation" OR "sensory sensitivity") AND TS=("gastrointestinal diseases" OR "gastrointestinal symptoms") AND TS=("gut-brain axis" OR "microbiota-gut-brain axis")	English language; publication years 2011-2026; no geographical restriction	Records retrieved: n=180
Embase	('autism spectrum disorder'/exp OR 'autism spectrum disorder':ti,ab,kw OR ASD:ti,ab,kw) AND ('gastrointestinal microbiome'/exp OR 'gut microbiota':ti,ab,kw OR 'intestinal microbiome':ti,ab,kw OR 'microbial dysbiosis':ti,ab,kw) AND ('sensory processing':ti,ab,kw OR 'sensory processing dysfunction':ti,ab,kw OR 'sensory dysregulation':ti,ab,kw OR 'sensory sensitivity':ti,ab,kw) AND ('gastrointestinal disease'/exp OR 'gastrointestinal symptoms':ti,ab,kw) AND ('gut brain axis'/exp OR 'gut-brain axis':ti,ab,kw OR 'microbiota-gut-brain axis':ti,ab,kw) AND [english]/lim AND 2011-2026	English language; publication years 2011-2026; no geographical restriction	Records retrieved: n=260
Google Scholar	("autism spectrum disorder" OR ASD) AND ("gut microbiota" OR "intestinal microbiome" OR "microbial dysbiosis") AND ("sensory processing" OR "sensory processing dysfunction" OR "sensory dysregulation" OR "sensory sensitivity") AND ("gastrointestinal diseases" OR "gastrointestinal symptoms") AND ("gut-brain axis" OR "microbiota-gut-brain axis")	English-language publications; publication years 2011-2026; sorted by relevance; used as a supplementary secondary source	First 200 records screened; potentially relevant records retrieved: n=92

Only English-language studies published from 1 January 2011 to 28 May 2026 were searched. There were no geographical constraints. Relevance to the review topic and objectives was evaluated for inclusion during the screening of the titles, the abstracts, and the full texts, but not as an electronic database filter.

Simple combinations of the main keywords were used to search Google Scholar as a secondary source. The first 200 results with relevance were filtered. All the studies included and all relevant review articles included in their reference lists were also hand searched to look for any other eligible studies not identified in the electronic database searches.

All retrieved data were entered in Rayyan (Rayyan Systems Inc., Cambridge, Massachusetts, United States), an AI-supported systematic review management platform. To avoid duplicate records, screening was preceded by duplicates being identified and eliminated. The titles and abstracts of potentially eligible studies were independently screened by two reviewers, and then the full texts of these studies were assessed. Any disagreements were resolved through discussion and consensus.

Eligibility Criteria

Studies published from 2011 to 2026 were included if they had participants with a diagnosis of ASD and measured at least one of the outcomes related to gastrointestinal symptoms, sensory dysregulation, or gut microbiota composition. Gastrointestinal side effects were constipation, diarrhea, abdominal pain, abdominal bloating, or other gastrointestinal symptoms reported clinically. Hyper- or hyporesponsiveness to auditory, visual, tactile, olfactory, or gustatory stimuli was a sensory outcome.

Studies that included non-ASD neurodevelopmental or clinical groups were included only when the non-ASD groups were clearly specified as a comparator group within the study, which also included an ASD group. Studies that examined only the control group without ASD were not included. If comparator groups were indicated, the results of the ASD sample were isolated and analyzed independently to keep the synthesis specific to ASD.

Observational studies (cross-sectional, case-control, cohort, and longitudinal studies) and interventional studies (randomized controlled and non-randomized trials) were included as eligible study designs. Studies were excluded if they did not contain a population of ASD, if they did not report the gastrointestinal, sensory, or gut microbiota outcomes, or if the design was not eligible (e.g., reviews, systematic reviews, meta-analyses, commentaries, editorials, conference abstracts without adequate data, and case reports).

Only studies published in full text and English were included. There were no geographical limitations imposed. Studies were also discarded if they did not report enough data to allow extraction and/or synthesis to be made. The publication restriction for primary studies that were eligible for the systematic evidence synthesis was in effect for the 2011-2026 period. In addition to the above, publications prior to and beyond this time period were consulted for historical, conceptual, or methodological background, and systematic reviews, narrative reviews, and methodological papers were consulted. The study characteristics tables, risk of bias assessment, and synthesis of findings from the included studies did not include these sources.

Study Selection Process

All the retrieved records were imported into Rayyan, and duplicate records were deleted prior to screening. All titles and abstracts were screened by two reviewers, F.B. and A.W., independently with predefined eligibility criteria. Both reviewers advanced records they considered to have the potential to be full text for further review. Full texts of potentially eligible studies were subsequently independently reviewed by the same two reviewers. Rayyan was used to record screening decisions, but they were only compared once each reviewer had reached that point. Any disagreements were resolved through discussion and consensus. A third reviewer was consulted when consensus could not be reached.

Data Extraction

A standardized data extraction form was created to ensure consistency and accuracy in the information that was collected from eligible studies. Two reviewers independently extracted data, minimizing bias and extraction errors. The data retrieved included the name of the author, year published, country where the study took place, study design, number of participants, participant characteristics, age range, diagnostic criteria for ASD, methods of assessment of the microbiota, gastrointestinal findings, sensory processing outcomes, metabolomic findings, and key conclusions. The reviewers identified the sensory construct assessed (e.g., sensitivity, feeding, swallowing) as well as the name of the instrument if it was reported, the caregiver, clinician, or respondent, and, if reported, whether the outcome was a caregiver report, a clinician assessment, a self-report, or an objective procedure. Sensory domains consisted of sensory over-responsivity, hyperreactivity, hyporeactivity, oral sensory sensitivity, food-related sensory responses, disgust sensitivity, and sensory profiles that were not limited to a specific sensory domain. If the instrument was not clearly mentioned in the available article or publication for an original study, it was not assumed but rather was written as not specified.

The reviewers identified the biological sample, laboratory technique, microbial domain targeted, taxonomic or functional resolution, and host-, inflammatory, or metabolomic-derived measurements related to the microbiome outcomes. The microbiome methods used included culture-based analysis, targeted quantitative polymerase chain reaction (qPCR), 16S ribosomal ribonucleic acid (rRNA) amplicon sequencing, shotgun metagenomic sequencing, fungal internal transcribed spacer sequencing, fecal metabolomics, biopsy-based mucosal analysis, and combined multi-omics analysis.

Risk of Bias Assessment

Validated assessment tools were employed for the methodological quality and risk of bias determination of the included studies, depending on their design. The Newcastle-Ottawa Scale (NOS) was used to assess the quality of the observational studies based on the selection of participants, the comparability of the study groups, and the outcome assessment (Table 2).

**Table 2:** NOS risk of bias in observational studies NOS: Newcastle-Ottawa Scale

Study	Selection criteria	Comparability	Outcome evaluation	Total NOS score	Revised quality
Mazurek et al., 2013 [19]	3	0	2	5	Moderate
Restrepo et al., 2025 [20]	3	1	2	6	Good
Aziz-Zadeh et al., 2025 [21]	3	1	2	6	Good
Chistol et al., 2018 [22]	3	0	2	5	Moderate
Tomova et al., 2020 [23]	3	0	2	5	Moderate
Yap et al., 2021 [24]	3	0	2	5	Moderate
Wu et al., 2025 [25]	3	0	2	5	Moderate
Adams et al., 2011 [26]	3	0	2	5	Moderate
Williams et al., 2011 [27]	3	0	2	5	Moderate
Kang et al., 2013 [28]	3	0	2	5	Moderate
De Angelis et al., 2013 [29]	3	0	2	5	Moderate
Tomova et al., 2015 [30]	3	0	2	5	Moderate
Strati et al., 2017 [31]	3	0	2	5	Moderate
Coretti et al., 2018 [32]	3	0	2	5	Moderate
Dan et al., 2020 [33]	3	0	2	5	Moderate
Vernocchi et al., 2022 [34]	3	0	2	5	Moderate
Li et al., 2023 [35]	3	0	2	5	Moderate
De Sales-Millán et al., 2024 [36]	3	0	2	5	Moderate
Su et al., 2024 [37]	3	1	2	6	Good
Khalil et al., 2021 [38]	3	0	2	5	Moderate

**Figure 1:**
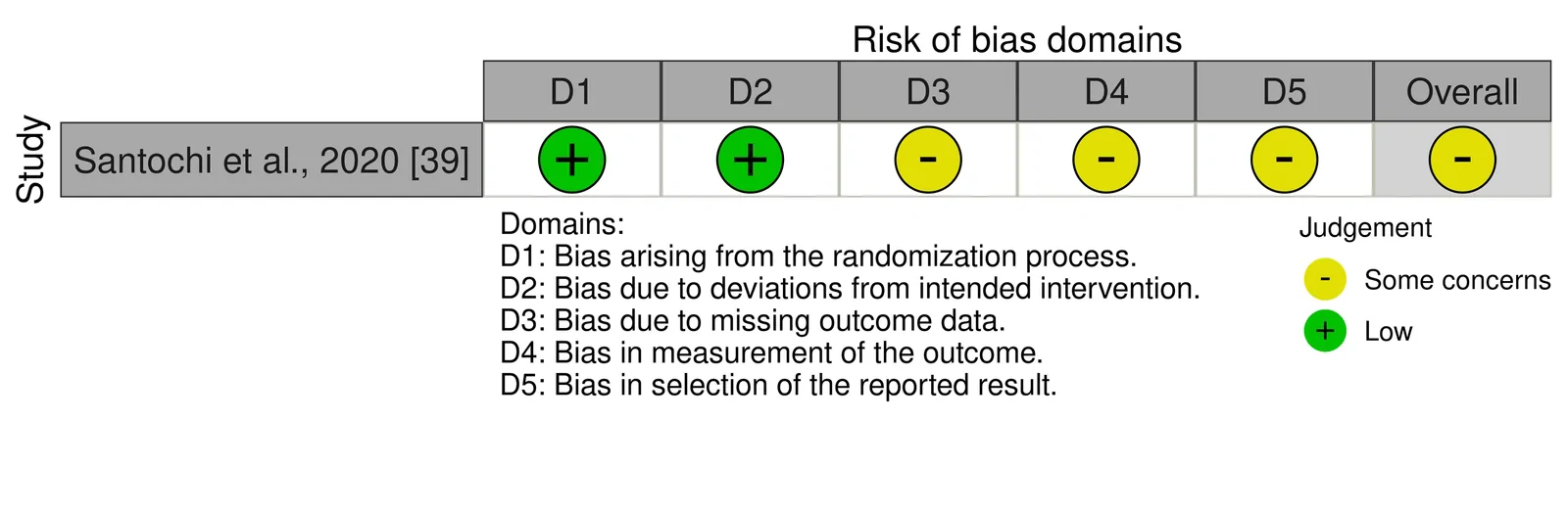
Risk of bias assessment of the included randomized controlled studies using the Cochrane RoB 2 tool

The Cochrane RoB 2 tool was used to assess randomized controlled trials (Table 3 and Figure 1) and the ROBINS-I V2 to assess the non-randomized intervention study (Table 4).

**Table 3:** Randomized controlled trials: Cochrane RoB 2 quality appraisal

Study	Randomization process	Deviations from intended interventions	Missing outcome data	Measurement of outcome	Selection of reported result	Overall risk of bias
Santocchi et al., 2020 [39]	Low	Low	Some concerns	Some concerns	Some concerns	Some concerns (primary outcome); high (gastrointestinal/sensory subgroup)

**Table 4:** Non-randomized controlled trials: ROBINS-I V2 quality appraisal

Study	Bias due to confounding	Classification of intervention	Selection of participants	Missing data	Measurement of outcomes	Selection of reported results	Overall risk of bias
Kang et al., 2017 [40]	High	Low	High	Low	High	Moderate	High
Wong et al., 2026 [41]	Low	Low	Low	Moderate	Moderate	Moderate	Low

Confounding was particularly addressed, as certain clinical factors, such as age, food selectivity, medication administration, exposure to antibiotics, stool consistency, gastrointestinal symptom status, and the presence of other gastrointestinal disorders, could affect the results of microbiome studies in ASD. Thus, even if observational studies were acceptable in the selection and outcome domains, but refused to earn full marks on the NOS domain, they were considered to have moderate rather than good methodological quality. Studies that got 1 comparability point and a total NOS score of 6 were considered good quality. None of the observational studies were judged to be excellent quality.

The risk of bias assessment included the following: definition or selection of study groups, comparability of study groups, assessment of exposures and outcomes, completeness of follow-up, control of confounding, missing data, randomization procedures (where applicable), and selective reporting of outcomes. Judgments were based on the criteria outlined in each of the assessment tools, not only on sample size or study design. Two reviewers, separately, evaluated each study. When differences arose, they were discussed and agreed upon, and a third reviewer was consulted when there was no consensus.

Data Synthesis

There was significant variation among the studies included in the analysis in the study design, participant characteristics, biological specimen, method of microbiome assessment, sequencing platform, genomic region analyzed, taxonomic resolution, functional analysis, and sensory outcome measurement. These microbiome methods covered culture-based analysis, targeted qPCR, 16S rRNA amplicon sequencing, shotgun metagenomics, fungal profiling, fecal metabolomics, phage sequencing, and biopsy-based mucosal analyses. These methods were not considered to be directly interchangeable as they measure different aspects of the intestinal ecosystem.

Culture-based methods are mainly used to detect organisms that grow under the conditions used in the laboratory, while targeted qPCR quantifies only predefined taxa or genes. However, in contrast, 16S rRNA sequencing can yield a more comprehensive coverage of the bacterial community with limited species and functional level resolution. While metabolomics looks at the biochemical products, shotgun metagenomics offers a more detailed species and functional information. Biopsy-based methods identify the characteristics of mucosa-associated microbial communities, which can be different from fecal communities. Therefore, statistical pooling of taxonomic/functional results was deemed inappropriate. Results were synthesized in a narrative and analyzed within categories that are, to the extent possible, similar in methodology.

Evidence sources were identified by publication type. The systematic synthesis and the study characteristics tables and risk of bias evaluations included only studies that met the eligibility criteria and were published in the prespecified time frame. Background and contextual interpretation from systematic reviews, meta-analyses, narrative reviews, methodological analyses, and publications outside the review period were not considered as primary empirical evidence.

Ethical Considerations

This study involved secondary data, and the literature has been used for the purpose of this study. No direct recruitment of human participants or primary data was collected; hence, there was no need for ethical approval and informed consent. However, all studies included were deemed to have been carried out within the ethical guidelines and regulations of the respective institutions and research ethics committees.

Results

**Figure 2:**
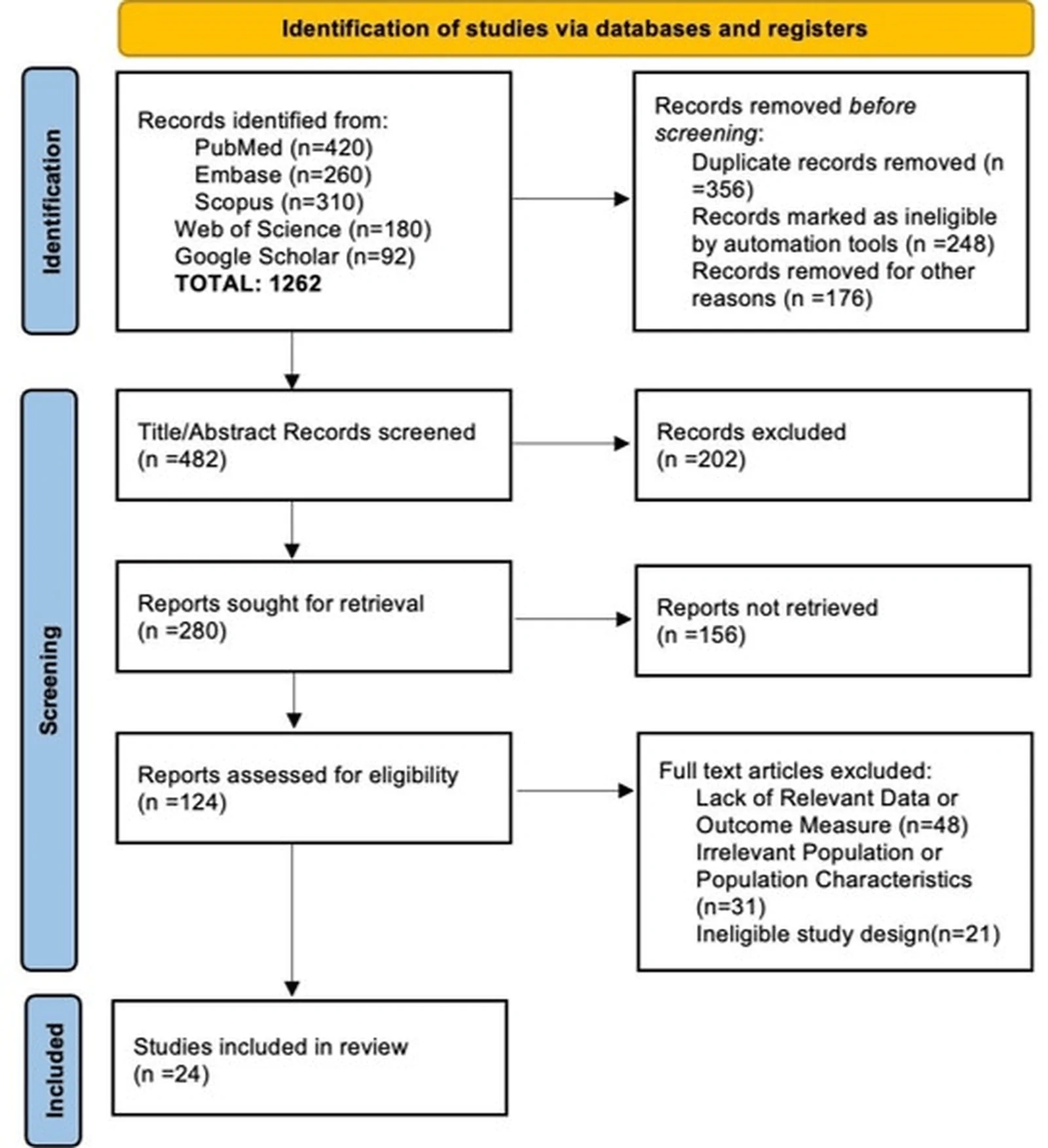
PRISMA flowchart of the study selection process PRISMA: Preferred Reporting Items for Systematic Reviews and Meta-Analyses

A total of 1,262 records were identified by the search strategy employed through systematic database searches across PubMed/MEDLINE, Scopus, Web of Science, Embase, and Google Scholar. A total of 482 records were screened after the removal of 356 duplicate records, 248 records marked as ineligible for screening by automated tools, and 176 records for other reasons. During the primary screening stage, the relevance of each article was assessed based on both titles and abstracts with regard to the research question, and 202 records were excluded. A total of 280 articles were requested for retrieval; however, 156 reports were not retrieved. Hence, only 124 reports were assessed for eligibility and were fully reviewed according to the inclusion criteria. The majority of these did not meet the inclusion criteria and were further excluded for specific reasons: 48 reports lacked relevant data or outcome measures, 31 reports involved an irrelevant population or population characteristics, and 21 reports had an ineligible study design. Finally, after a detailed full-text assessment against the prespecified eligibility criteria, 24 studies fulfilled the inclusion criteria and were incorporated in the systematic review, which was the basis for the analysis and conclusions of the study, as illustrated in the PRISMA flow diagram (Figure 2).

Characteristics of the Included Studies

The 24 studies investigated different aspects of ASD and gastrointestinal symptoms, sensory features, dietary patterns, and gut microbiota. Most of the studies included were cross-sectional, and a few were interventional and longitudinal. Based on the above, the overall evidence supports associations, and there is limited evidence on temporal and/or causal relationships. Sample size varied from studies of less than 50 children to those with more than 1,600 children, with most studies conducted in the United States, China, Italy, and other countries. In terms of methods, the area of research starting to develop from parent-reported symptoms and culture-based microbial analysis to advanced technologies, such as 16S rRNA sequencing, shotgun metagenomics, fecal metabolomics, and microbiota transfer therapy, has grown with an interest in multi-omics and intervention to understand the gut-brain axis in ASD. The detailed characteristics of all included studies are presented in Table 5.

**Table 5:** Characteristics of the included studies Where an exact instrument or reporter was not described in the available study report, this is explicitly indicated rather than inferred. ASD: autism spectrum disorder; TD: typically developing; GI: gastrointestinal; fMRI: functional magnetic resonance imaging; SCFA: short-chain fatty acids; PDD-NOS: pervasive developmental disorder-not otherwise specified; FMT: fecal microbiota transplantation; CARS: Childhood Autism Rating Scale; SSP: Short Sensory Profile; ADOS: Autism Diagnostic Observation Schedule; GSI: Gastrointestinal Severity Index; OCD: obsessive-compulsive disorder

Study	Sensory or related construct	Instrument or assessment approach	Reporter/assessor	Measurement type
Mazurek et al., 2013 [19]	Sensory over-responsivity, anxiety, chronic GI symptoms	Sensory over-responsivity and anxiety measures; chronic GI problems recorded in the Autism Treatment Network dataset	Parent/caregiver-reported clinical network data	Reported/observational
Wong et al., 2026 [41]	Sensory hyperresponsiveness, anxiety, abdominal pain	Symptom assessments conducted during a 12-week open-label synbiotic study; exact sensory scale not specified in the available report	Participant caregiver/clinical study team; exact source not specified	Repeated clinical outcome assessment
Restrepo et al., 2025 [20]	Sensory problems, behavioral symptoms, sleep, GI symptoms	Nine GI symptoms assessed using a physician-administered parent interview; sensory instrument not specified in the available report	Physician-administered parent interview and behavioral assessments	Clinician-facilitated caregiver report
Aziz-Zadeh et al., 2025 [21]	Sensory sensitivities, disgust sensitivity, ASD severity	Behavioral assessments, ADOS scores, socio-emotional and sensory fMRI paradigms	Clinician/participant behavioral assessment and objective neuroimaging	Reported plus objective
Chistol et al., 2018 [22]	Oral sensory processing and food selectivity	Oral sensory processing and food selectivity assessment; exact scale name not specified in the available report	Parent/caregiver-reported assessment	Standardized or structured report; exact tool not specified
Tomova et al., 2015 [30]	Food selectivity and dietary intake	Dietary and food habit assessment; no direct standardized sensory scale reported	Parent/caregiver dietary reporting	Dietary proxy for sensory-related food selectivity
Yap et al., 2021 [24]	Restricted interests, dietary preferences, dietary diversity	Autism-related traits, dietary intake, and stool characteristics; exact sensory scale not specified	Participant/caregiver phenotypic and dietary data	Behavioral and dietary assessment
Wu et al., 2025 [25]	Atypical eating behavior, diet quality, ASD symptoms, GI complications	Granular dietary assessment, nutrient indices, additive exposure, and phenotypic profiles	Participant/caregiver-reported phenotypic and dietary data	Dietary and behavioral assessment
Adams et al., 2011 [26]	GI symptom severity and autism severity	Modified six-item GSI and Autism Treatment Evaluation Checklist	Parent/caregiver-reported questionnaires	Standardized/modified questionnaires
Williams et al., 2011 [27]	GI disease and behavioral impairment	Clinical GI evaluation; no direct sensory assessment reported	Clinical assessment	Clinician-rated/biological
Kang et al., 2013 [28]	Autism severity and GI symptoms	Survey of autism severity and GI symptoms; exact instruments not specified in the available report	Parent/caregiver-reported survey	Reported outcome
De Angelis et al., 2013 [29]	ASD/PDD-NOS clinical status; no direct sensory outcome	Clinical group classification; no standardized sensory measure reported	Clinical assessment	Not applicable for sensory measurement
Tomova et al., 2015 [30]	Autism severity, restricted/repetitive behavior, GI dysfunction	Autism Diagnostic Interview restricted/repetitive behavior subscale and GI severity assessment	Clinician-administered diagnostic assessment and reported GI symptoms	Clinician-rated/reported
Strati et al., 2017 [31]	ASD severity, constipation, and GI status	Clinical ASD severity and constipation classification; no direct sensory scale reported	Clinical/participant data	Clinical and reported
Coretti et al., 2018 [32]	ASD clinical status; no direct sensory assessment	No direct sensory instrument reported	Not applicable	Not applicable
Dan et al., 2020 [33]	ASD, constipation, and GI subgroup status	Clinical and GI subgroup classification; no direct sensory instrument reported	Clinical assessment	Clinical
Vernocchi et al., 2022 [34]	Food selectivity, picky eating, ASD severity, and GI symptoms	Clinical and dietary phenotyping; exact sensory instrument not specified	Caregiver/clinical assessment	Reported and clinical
Li et al., 2023 [35]	ASD clinical status; no direct sensory assessment	No direct sensory instrument reported	Not applicable	Not applicable
De Sales-Millán et al., 2024 [36]	ASD clinical status; no direct sensory assessment	No direct sensory instrument reported	Not applicable	Not applicable
Su et al., 2024 [37]	Extensive ASD phenotype data; sensory tool not specified	Phenotypic assessment accompanying metagenomic analysis	Clinical/caregiver data; exact source not specified	Phenotypic
Kang et al., 2017 [40]	GI symptoms and ASD behavioral symptoms	Gastrointestinal Symptom Rating Scale and clinical ASD assessments	Participant caregivers and clinical assessors	Reported and clinician-rated
Khalil et al., 2021 [38]	Sensory impairment, autism severity, and GI symptoms	SSP, CARS, and modified six-item GSI	Parent/caregiver SSP and GI report; clinician-rated CARS	Standardized reported and clinician-rated
Martínez-González et al., 2025 [42]	Sensory hyperreactivity, hyporeactivity, pain, GI symptoms, and OCD symptoms	Cross-disorder sensory, pain, GI, and OCD assessments; exact instrument names not specified in the available report	Participant/caregiver or clinician source not specified	Reported/clinical; exact source not reported
Santocchi et al., 2020 [39]	Sensory profile, adaptive functioning, GI symptoms, and ASD severity	Sensory profile assessment, ADOS-Calibrated Severity Score, and GI measures; exact sensory profile version not specified in the available report	Caregiver-reported sensory profile and clinician-administered ADOS	Reported and clinician-rated

Sensory and related outcome assessment

The studies included a range of measurement approaches for sensory and related behavioral outcomes. The constructs measured were sensory over-responsivity, hyperreactivity, hyporeactivity, oral sensory sensitivity, food selectivity, disgust sensitivity, and general sensory profiles. Khalil et al. measured sensory profile with the Short Sensory Profile (SSP), and Santocchi et al. measured sensory profile and clinician-administered ADOS outcomes [38,39]. Chistol et al. investigated oral sensory processing in the context of food refusal and vegetable consumption, but did not indicate the specific sensory instrument used in their study [22]. Sensory over-responsivity was evaluated in a large clinical network sample of caregivers reporting sensory problems by themselves by Mazurek et al. and by Restrepo et al., who included sensory problems along with physician diagnosis of gastrointestinal symptoms in a larger longitudinal sample [19,20].

Other studies used behavioral assessment, study-specific symptom measures, dietary proxies, or combined objective and/or reported outcomes. Aziz-Zadeh et al., for instance, used sensory and disgust-related behavioral measures, as well as task-based functional magnetic resonance imaging (fMRI), while Yap et al. and Wu et al. focused on restricted interests, atypical eating behavior, and dietary patterns as microbiome variance factors [21,24,25]. Due to the varied sensory construct, instrument, respondent, assessor, and measurement modality in the studies, direct comparisons of the findings (such as oral sensitivity, general sensory over-responsivity, hyperreactivity, and global sensory profile scores) were not made.

Heterogeneity of microbiome assessment methods

The evidence of the microbiome was obtained by significantly different biological and analytical approaches. Initially, stool culture, biochemical measurements, and qPCR were employed, while later, 16S rRNA sequencing, shotgun metagenomics, metabolomics, and multi-kingdom analyses were increasingly used. Adams et al. analyzed cultivable bacteria and yeast along with fecal short-chain fatty acids (SCFAs) and digestive markers, and Tomova et al. utilized targeted qPCR to quantify certain groups of bacteria [26,30]. In contrast, Kang et al. analyzed 16S pyrosequences of V2 and V3, Vernocchi et al. V3-V4 targeted sequencing with inflammatory and permeability markers, and Yap et al. shotgun stool metagenomics [24,34,40].

Other studies quantified non-equivalent related biological domains. Aziz-Zadeh and colleagues measured fecal metabolites and their relationships with fMRI activity instead of the microbial communities themselves [21]. Williams and colleagues examined mucosa-associated (not fecal) microbial environments by analyzing mucosal biopsies and intestinal gene expression [27]. Multi-kingdom shotgun sequencing was employed by Su et al. to assess bacterial, fungal, archaeal, viral, gene, and pathway markers and offered significantly greater resolution than 16S bacteria-only studies [37]. Thus, the individual taxa or functional pathways detected by different platforms were not nationalized as being directly comparable or as being a single reproducible microbial signature (Table 6).

**Table 6:** Microbiome, metabolomic, and related biological methods used in the included studies PCR: polymerase chain reaction; rRNA: ribosomal ribonucleic acid; LC: liquid chromatography; MS: mass spectrometry; SCFA: short-chain fatty acid; fMRI: functional magnetic resonance imaging; OTUs: Operational Taxonomic Units; KEGG: Kyoto Encyclopedia of Genes and Genomes

Study	Biological material	Laboratory/analytical method	Primary analytical level	Important comparability consideration
Mazurek et al., 2013 [19]	None	No microbiome testing	Clinical symptom association	Does not provide microbial composition data
Wong et al., 2026 [41]	Stool	Shotgun metagenomics and metabolomics	Species, functional pathways, and metabolites	Open-label intervention; repeated multi-omics data
Restrepo et al., 2025 [20]	None	No microbiome analysis	Longitudinal clinical phenotyping	Gastrointestinal and sensory findings cannot identify microbial mechanisms
Aziz-Zadeh et al., 2025 [21]	Fecal samples	Fecal metabolomics combined with task-based fMRI	Microbial-related metabolites and brain activity	Measures metabolites rather than microbial taxa directly
Chistol et al., 2018 [22]	None	No microbiome analysis	Sensory and dietary outcomes	No microbial composition assessment
Tomova et al., 2015 [30]	Stool/fecal DNA	High-throughput 16S-based sequencing	Bacterial diversity and relative taxonomic abundance	Relative composition; affected by sequencing region and pipeline
Yap et al., 2021 [24]	Stool	Shotgun metagenomics	Species-level taxonomic and functional profiling	Higher resolution than 16S; controlled extensively for diet and stool characteristics
Wu et al., 2025 [25]	Stool	Fecal shotgun metagenomics with detailed dietary assessment	Species-level microbiome and diet-microbiome networks	Findings integrate diet and phenotypic exposures
Adams et al., 2011 [26]	Stool	Bacterial and yeast culture, SCFA analysis, immune and digestive markers	Cultivable organisms and biochemical markers	Culture detects only organisms growing under selected conditions
Williams et al., 2011 [27]	Ileal/intestinal biopsy	Mucosal bacterial metagenomics and host gene-expression analysis	Mucosa-associated microbiota and intestinal host function	Biopsy microbiota is not directly comparable with fecal microbiota
Kang et al., 2013 [28]	Fecal DNA	16S rDNA V2/V3 pyrosequencing	Bacterial diversity and genus-level relative abundance	Amplicon region and older pyrosequencing platform affect comparability
De Angelis et al., 2013 [29]	Stool	16S rDNA/rRNA amplicon pyrosequencing, culture-dependent analysis, and fecal metabolomics	Total and metabolically active bacteria plus metabolites	Combined methods measure different biological components
Tomova et al., 2015 [30]	Stool	Targeted real-time quantitative PCR	Preselected bacterial taxa	Cannot characterize untargeted community-wide diversity
Strati et al., 2017 [31]	Stool	Bacterial 16S and fungal community profiling	Bacterial and fungal relative abundance	Multi-kingdom analysis differs from bacteria-only studies
Coretti et al., 2018 [32]	Stool	16S rRNA sequencing, SCFA assessment, and functional inference	Bacterial OTUs, predicted functions, and metabolites	Predicted functions are not equivalent to directly measured metagenomic pathways
Dan et al., 2020 [33]	Stool	16S rRNA sequencing; shotgun metagenomics in a constipation subgroup; LC-MS metabolomics	Community composition, species/functions, and metabolites	Different methods were applied to different sample subsets
Vernocchi et al., 2022 [34]	Stool	16S V3-V4 targeted metagenomics, inferred pathways, inflammation and permeability markers	Bacterial composition, predicted function, and host markers	Predicted functional profiles and fecal markers are method-dependent
Li et al., 2023 [35]	Stool	16S rRNA sequencing with KEGG pathway inference	Genus-level composition and predicted pathways	Functional findings were inferred rather than directly sequenced
De Sales-Millán et al., 2024 [36]	Stool	16S V3-V4 amplicon sequencing and predicted metabolic pathways	Bacterial composition and inferred functions	Limited species resolution; predicted functions are not direct metagenomic measurements
Su et al., 2024 [37]	Fecal samples	Shotgun multi-kingdom metagenomic sequencing and machine learning	Archaea, bacteria, fungi, viruses, genes, and pathways	Broad multi-kingdom resolution differs from 16S bacteria-only studies
Kang et al., 2017 [40]	Stool	Bacterial and phage deep sequencing before and after microbiota transfer therapy	Microbial diversity, engraftment, and virome changes	Non-randomized open-label intervention with high risk of bias
Khalil et al., 2021 [38]	Stool	Targeted quantitative real-time PCR for *C. difficile *and toxins A and B	Single targeted organism and toxin genes	Cannot be interpreted as whole-community microbiome profiling
Martínez-González et al., 2025 [42]	None	No microbiome analysis	Sensory, pain, and gastrointestinal comparison	Does not provide microbial composition data
Santocchi et al., 2020 [39]	Not described in supplied summary as a sequencing outcome	Probiotic randomized controlled trial	Clinical intervention outcomes	Clinical efficacy evidence should not be conflated with taxonomic profiling

Table 7 summarizes the studies that investigated gastrointestinal symptoms in conjunction with anxiety, sensory characteristics, dietary behavior, or overall behavioral measures in people with ASD. A few studies found that children with gastrointestinal symptoms had a high prevalence or higher severity of gastrointestinal symptoms, and others found that gastrointestinal symptoms were related to anxiety, sensory over-responsivity, internalizing symptoms, sleep disturbance, or behavioral impairment. But sensory outcomes were not measured consistently across studies. Some investigations employed sensory-specific instruments, some relied on food selectivity, some used structured eating assessments, and others relied on general clinical instruments to infer sensory-related difficulties.

**Table 7:** GI symptoms, anxiety, and sensory over-responsivity in ASD GI: gastrointestinal; ASD: autism spectrum disorder; fMRI: functional magnetic resonance imaging; ADHD: attention-deficit/hyperactivity disorder

Study	Sample	Main GI findings	Anxiety/sensory-related finding	Sensory outcome directly measured?	Nature of sensory assessment	Key association
Mazurek et al., 2013 [19]	2,973 individuals with ASD	Chronic GI problems were reported in approximately 24% of participants	Anxiety and sensory over-responsivity were both associated with chronic GI problems	Yes	Sensory over-responsivity was directly assessed through caregiver-reported clinical measures	Anxiety and sensory over-responsivity independently predicted chronic GI problems
Restrepo et al., 2025 [20]	322 children with ASD	GI symptoms were more frequent, multiple, and persistent over time	GI symptoms were linked with internalizing problems, sensory difficulties, and sleep disturbance	No/not clearly specified	Sensory problems were reported as part of broader clinical or behavioral assessment; the exact sensory instrument was not specified	Persistent GI symptoms were associated with greater behavioral and functional impairment
Aziz-Zadeh et al., 2025 [21]	43 participants with ASD	The study focused primarily on fecal metabolites rather than direct GI symptom prevalence	Tryptophan-related metabolites were associated with sensory sensitivities and activity in the insula and cingulate cortex	Yes	Sensory sensitivities were assessed behaviorally and examined alongside task-based fMRI	Metabolite, sensory, and brain activity findings were correlational and did not establish mediation or causality
Chistol et al., 2018 [22]	53 children with ASD	Direct GI symptoms were not the principal outcome	Oral sensory sensitivity was associated with food refusal and lower vegetable intake	Yes	Oral sensory processing was directly assessed, but this was a domain-specific measure rather than a comprehensive sensory profile	Supports an oral sensory sensitivity-food selectivity association
Adams et al., 2011 [26]	58 children with ASD	GI symptom severity was higher in the ASD group	No direct sensory outcome was reported	No	Sensory processing was not assessed	GI symptom severity correlated with autism severity, reported as r=0.59
Wu et al., 2025 [25]	462 participants with ASD	Greater GI complications were reported in association with poorer dietary quality	Atypical eating behavior was examined, but direct sensory processing was not assessed	No	Dietary and eating behavior measures were used as indirect proxies; these are not equivalent to direct sensory assessment	Poor diet quality and food-additive exposure were associated with GI and microbiome variation
Vernocchi et al., 2022 [34]	41 children with ASD	Approximately 73% had GI symptoms	No direct sensory outcome was reported	No	Sensory processing was not directly assessed	GI status was an important factor associated with microbiome composition
Khalil et al., 2021 [38]	58 children with ASD	GI dysfunction was common, but no significant association with *Clostridium difficile *was identified	Sensory impairment was common	Yes	Sensory processing was directly measured using the Short Sensory Profile	Sensory and GI difficulties co-occurred, but *C. difficile *was not specifically associated with ASD-related GI symptoms
Martínez-González et al., 2025 [42]	37 participants with ASD	Abdominal GI symptoms were more severe in ASD than in ADHD and learning disability groups	Greater sensory hyperreactivity was reported in ASD	Yes, but exact instrument not specified	Sensory hyperreactivity was directly compared across diagnostic groups; the specific tool was not clearly identified in the available report	Supports a co-occurring sensory-GI symptom profile in ASD without establishing causality
Santocchi et al., 2020 [39]	85 children with ASD	No overall primary treatment effect was established; selected GI improvements were reported, particularly in the GI subgroup	Sensory profile improvement was reported in a subgroup	Yes	A caregiver-reported sensory profile was used alongside clinician-administered ASD outcomes	Subgroup findings suggested possible GI, adaptive, and sensory benefits, but these were exploratory and should be interpreted cautiously

Some studies linked oral sensory sensitivity with food refusal and decreased food variety, but these studies only focused on the sensory association of a single food domain. Most studies have been observational and cross-sectional, making it unclear what direction the relationship is between gastrointestinal symptoms and anxiety and sensory difficulties. In subgroup analyses, Santocchi et al. reported improvement in gastrointestinal and sensory measures, though no overall primary effect of the treatment was proven [39]. Based on the current evidence, there is a possible association between gastrointestinal dysfunction, anxiety, and sensory features in ASD, but the available evidence does not support that one will directly cause the other or that treating one will directly improve the other or that one will worsen the other.

Table 8 summarizes the microbiome findings together with the principal methodological and confounding considerations reported across the included studies. The findings should not be interpreted as demonstrating a consistent ASD-specific microbial profile because the studies differed substantially in participant characteristics, specimen processing, microbiome platform, sequencing resolution, dietary assessment, gastrointestinal status, stool consistency, medication exposure, and control of antibiotic or probiotic use. Culture-based analysis, 16S sequencing, shotgun metagenomics, multi-kingdom sequencing, and functional prediction also produce different types of data and are not directly interchangeable.

**Table 8:** Gut microbiota findings and methodological limitations in ASD studies "Not reported" indicates that the relevant information was not clearly available in the extracted study information and does not confirm that the factor was not measured. The F/B ratio is presented only where reported by the original study and is not treated as a reliable standalone indicator of dysbiosis. ASD: autism spectrum disorder; F/B ratio: Firmicutes-to-Bacteroidetes ratio; KEGG: Kyoto Encyclopedia of Genes and Genomes; SCFA: short-chain fatty acid; 16S rRNA: 16S ribosomal ribonucleic acid; GI: gastrointestinal

Study	Microbiome method used	α-Diversity	F/B ratio	Key reported taxonomic findings	Stool consistency controlled?	Diet controlled/assessed?	Antibiotic/probiotic exposure controlled?	Main limitation
Kang et al., 2013 [28]	Fecal 16S rDNA V2/V3 pyrosequencing	Decreased	Not reported	Lower *Prevotella*, *Coprococcus*, and unclassified *Veillonellaceae*	Not reported	Dietary patterns were assessed, but comprehensive statistical control was not clearly reported	Not reported	Small sample; most ASD participants had GI symptoms; limited control of diet, stool characteristics, and clinical confounders
Adams et al., 2011 [26]	Stool bacterial and yeast culture with SCFA and digestive marker analysis	Not assessed using community-wide sequencing	Not reported	Higher cultured *Lactobacillus *and lower cultured *Bifidobacterium*	Not reported	Fiber intake was discussed, but dietary adjustment was not clearly reported	Probiotic use was recorded, but residual confounding remained	Culture-based findings are not directly comparable with sequencing studies; probiotic use may have influenced results
Strati et al., 2017 [31]	Fecal 16S rRNA sequencing with fungal community profiling	Not clearly reported as a consistent group-level change	Increased, primarily due to reduced *Bacteroidetes*	Higher *Collinsella*, *Corynebacterium*, *Dorea*, and *Lactobacillus*; lower *Alistipes*, *Bilophila*, *Dialister*, *Parabacteroides*, and *Veillonella*	Constipation was examined, but stool consistency adjustment was not clearly specified	Not reported	Not reported	Findings may have been influenced by constipation, diet, ASD severity, and combined bacterial-fungal analysis
Coretti et al., 2018 [32]	Fecal 16S rRNA sequencing, SCFA measurement, and predicted functional analysis	Not reported	Not reported	Higher *Faecalibacterium prausnitzii *and lower *Bifidobacterium longum*	Not reported	Not reported	Not reported	Small, age-restricted cohort; predicted functions are not equivalent to directly measured metagenomic pathways
Li et al., 2023 [35]	Fecal 16S rRNA sequencing with KEGG pathway inference	Decreased	Not reported	Higher *Faecalibacterium*, *Blautia*, *Eubacterium eligens *group, *Parasutterella*, and *Veillonella*; lower *Prevotella 9 *and *Agathobacter*	Not reported	Not reported	Not reported	Cross-sectional design; residual confounding; functional pathways were inferred rather than directly measured
Su et al., 2024 [37]	Fecal multi-kingdom shotgun metagenomics with machine learning analysis	Not summarized as a single overall diversity result	Not used as a principal outcome	Multiple bacterial, fungal, archaeal, viral, gene, and pathway markers differed between groups	Specific adjustment should be verified from the full study	Extensive phenotypic data were collected, but precise dietary adjustment should be verified from the full study	Specific adjustment should be verified from the full study	Cross-sectional diagnostic modelling; machine learning discrimination does not establish causality or clinical validity
Yap et al., 2021 [24]	Fecal shotgun metagenomics	Reduced diversity was associated mainly with restricted dietary variety rather than a strong direct ASD effect	Not treated as a principal marker	Limited direct ASD-related taxonomic differences; *Romboutsia timonensis *was among the few reported direct signals	Yes; stool consistency was explicitly analyzed	Yes; dietary intake and dietary diversity were extensively assessed	Not clearly reported in the available extracted information	Cross-sectional design; supports a diet- and stool-related explanatory model but cannot establish mediation or causality

Several studies reported differences in α-diversity or taxonomic composition, but the direction and magnitude of these findings were inconsistent. Reduced α-diversity was reported in some cohorts, including Kang et al. and Li et al., whereas other studies did not report a comparable overall reduction or used methods that did not permit community-wide diversity assessment [28,35]. Yap et al. found that dietary diversity and stool consistency were more strongly associated with microbiome variation than ASD diagnosis itself, indicating that dietary and gastrointestinal factors may partly explain apparent group differences [24].

Individual taxa such as *Prevotella*, *Bifidobacterium*, *Lactobacillus*, *Faecalibacterium prausnitzii*, and *Blautia* were reported as increased or decreased in selected studies, but these patterns were not consistently replicated across cohorts. Accordingly, these findings should be regarded as study-specific and method-dependent rather than as a reproducible taxonomic signature of ASD.

The *Firmicutes*-to-*Bacteroidetes *ratio was reported in only a limited subset of studies and was not used as a reference standard in this review. Because this ratio combines highly diverse bacterial groups into a single summary measure and may vary with age, diet, stool consistency, gastrointestinal symptoms, geography, sequencing methodology, and bioinformatic processing, it has limited interpretive value when used alone. Overall, the available evidence indicates heterogeneous microbiome associations in ASD, with substantial potential influence from methodological differences and residual confounding.

Table 9 highlights the evidence supporting the potential for a clinically relevant pathway connecting sensory and behavioral characteristics to gut microbiome variation in ASD. Chistol et al. showed greater food refusal coupled with decreased intake of vegetables to be related to oral sensory sensitivity, confirming the first pathway of sensory dysregulation to dietary restriction of food exposure [22]. That research, however, didn't investigate the microbiome; thus, only the sensory sensitivity/food selectivity part of the proposed model is supported.

**Table 9:** Diet, food selectivity, sensory features, and microbiome associations in ASD "Not evaluated" indicates that the study did not examine the relevant microbiome or mediation outcome. "Not clearly established" indicates that the available study information did not show whether microbiome differences remained statistically significant after comprehensive dietary adjustment. Evidence for dietary mediation should be interpreted cautiously unless a formal mediation model, temporal sequence, and adequate confounder control were demonstrated. Food selectivity or atypical eating behavior should not automatically be treated as equivalent to direct measurement of sensory processing. ASD: autism spectrum disorder

Study	Food selectivity or dietary feature	Direct ASD-microbiome association supported?	Diet supports a mediating/explanatory pathway?	Sensory-driven food selectivity influences diet?	Microbiome differences remain after dietary adjustment?	Main scientific interpretation
Chistol et al., 2018 [22]	Greater food refusal and reduced vegetable intake in children with atypical oral sensory sensitivity	Not evaluated; microbiome composition was not assessed	Not evaluated	Yes. Oral sensory sensitivity was directly associated with food refusal and restricted dietary variety	Not applicable	Supports the first stage of the proposed pathway: oral sensory sensitivity → food selectivity/restricted diet
Tomova et al., 2015 [30]	High food selectivity and differences in fiber, fat, protein, micronutrient, and fermented food intake	Partially supported. Group-level microbiome differences were reported, but dietary factors were also associated with microbiome variation	Yes, as a potential explanatory factor, although formal statistical mediation was not established	Indirectly supported. Food selectivity was assessed, but a direct sensory processing measure was not clearly reported	Not clearly established. The available information does not confirm whether all microbiome differences remained after comprehensive dietary adjustment	Supports an association between restricted dietary intake and microbiome composition, but not causal mediation
Yap et al., 2021 [24]	Restricted interests, reduced dietary diversity, and dietary preferences	Limited. Few direct ASD-microbiome associations remained after considering major covariates	Yes. Dietary preferences and reduced dietary diversity were proposed as major explanatory factors	Indirectly supported. Restricted interests and dietary preferences were examined, but comprehensive sensory processing was not directly measured	Largely attenuated after adjustment. Age, diet, and stool consistency were stronger correlates than ASD diagnosis	Provides the strongest support for the alternative pathway: ASD-related behavioral or sensory preferences → restricted diet → microbiome variation, without establishing causality
Wu et al., 2025 [25]	Atypical eating behavior, poorer diet quality, nutrient differences, and food-additive exposure	Partially supported. ASD-specific diet-microbiome interaction patterns were reported	Yes, as an explanatory pathway. Dietary quality and additive exposure were associated with microbiome organization	Indirectly supported. Atypical eating behavior may reflect sensory-related preferences, but direct sensory assessment was not clearly reported	Some associations remained in integrated analyses, although the extent of residual dietary and clinical confounding was not fully established	Suggests that dietary exposures may interact differently with the microbiome in ASD, but does not prove a direct ASD effect or formal mediation

Tomova et al. found correlations between food selectivity, nutrients consumed, dietary compositions, and microbiome characteristics [23]. The results suggest that diet may be a plausible explanation, but formal mediation and enduring microbiome changes following thorough dietary changes were not definitively linked. The most negative results of a direct association between ASD and the microbiome were found by Yap et al., who found little evidence of direct associations with ASD diagnosis, but found that dietary diversity, dietary preferences, and stool consistency were better correlates of microbiome variation [24]. However, the cross-sectional study design does not allow for causal inferences concerning the associations observed.

Wu et al. also found ASD-specific correlations between diet quality, atypical eating habits, nutrient exposure, food additives, and microbiome organization [25]. The results indicate a possible role for dietary exposures in microbiome variation in ASD, but they do not show that sensory-driven food selection was directly measured and no microbiome differences were found independently of all dietary and clinical confounders.

Together, this evidence suggests a viable line of sensory or behavioral differences leading to food selection or diet restriction and ultimately to microbiome variation. The level of support varies, however, across studies, and there is no evidence from observations that confirms temporal sequence, formal mediation, or causality. Microbiome differences not necessarily attributable to ASD should be differentiated from direct effects of the microbiome on the ASD diagnosis.

Table 10 summarizes the metabolomic and functional findings reported in the included ASD studies, their methodological characteristics, and their main limitations. Identified metabolites and pathways comprised tryptophan metabolites, SCFAs, butyrate-related functions, neurotransmitter-associated metabolic pathways, and microbial biosynthetic pathways. The results of these studies, however, were obtained under varying biological samples, analytical systems, participant groups, and study goals, which make direct comparisons between investigations difficult.

**Table 10:** Metabolites and functional pathways reported in ASD studies "Not clearly reported" indicates that sufficient methodological detail was unavailable in the extracted study information. Reported metabolite-brain or metabolite-behavior associations are correlational and should not be interpreted as evidence of causal mechanisms. GI: gastrointestinal; ASD: autism spectrum disorder; fMRI: functional magnetic resonance imaging; rRNA: ribosomal ribonucleic acid; SCFA: short-chain fatty acid; LC-MS: liquid chromatography-mass spectrometry; GABA: gamma-aminobutyric acid; KEGG: Kyoto Encyclopedia of Genes and Genomes

Study	Sample size	Biological sample	Analytical method	Principal metabolites/functional pathways	Clinical relevance	Brain/behavior association	Diet or GI symptoms adjusted?	Exploratory or hypothesis-driven?	Main limitation
Aziz-Zadeh et al., 2025 [21]	84 (43 ASD; 41 neurotypical)	Fecal samples with task-based fMRI	Fecal metabolomics, behavioral assessment, and fMRI	↓ kynurenate; altered indolelactate and tryptophan-related metabolites	Suggests altered tryptophan metabolism in ASD	Metabolites were associated with insular and cingulate activity, sensory sensitivity, and ASD severity	Not clearly reported	Exploratory, cross-sectional multimodal analysis	Correlational findings; causality cannot be inferred
Adams et al., 2011 [26]	97 (58 ASD; 39 controls)	Stool	Bacterial and yeast culture, SCFA analysis, digestive and inflammatory markers	↓ total SCFAs (acetate, propionate, valerate)	Indicates altered microbial metabolic activity	No direct brain or behavioral metabolic association evaluated	Probiotic use considered; comprehensive dietary adjustment not clearly reported	Hypothesis-driven observational study	Culture-based methods limit comparison with sequencing studies
Coretti et al., 2018 [32]	Not clearly reported in extracted data	Stool	16S rRNA sequencing, SCFA measurement, predicted KEGG functional analysis	↑ butyrate; enrichment of predicted butyrate production pathways	Suggests altered microbial functional capacity	No direct brain-behavior association reported	Not clearly reported	Exploratory observational study	Functional predictions were inferred rather than directly measured
Dan et al., 2020 [33]	143 for 16S analysis; metabolomic subgroup analyzed separately	Stool	16S sequencing, shotgun metagenomics (subgroup), LC-MS metabolomics	Altered neurotransmitter-related metabolic pathways (serotonin, dopamine, histidine, GABA)	Suggests microbiome-related metabolic dysregulation	No direct neuroimaging correlation reported	Constipation subgroup considered; full dietary adjustment not clearly reported	Exploratory multi-omics analysis	Cross-sectional design and subgroup analyses limit generalizability
Su et al., 2024 [37]	1,627 children	Stool	Shotgun multi-kingdom metagenomics with functional pathway analysis and machine learning modelling	↓ ubiquinol-7 biosynthesis; ↓ thiamine diphosphate biosynthesis; multiple microbial genes and pathways altered	Functional pathways contributed to diagnostic classification models	No direct brain imaging association examined	Extensive phenotype data available; exact dietary/GI adjustment should be verified from the original article	Primarily diagnostic and exploratory modelling	Diagnostic discrimination does not establish biological causality or clinical utility

Aziz-Zadeh et al. reported decreases in kynurenate and changes in indole lactate and other metabolites of tryptophan that were correlated with sensory sensitivities, the severity of ASD, and activity in the insular and cingulate cortices [21]. These conclusions are based on cross-sectional, exploratory, and non-causal studies and should not be considered as proof of a direct causal relationship between changes in brain function and behavioral symptoms and microbial metabolites.

Adams et al. found that several SCFAs were decreased, and Coretti et al. reported an increase in butyrate production and predicted an enrichment of butyrate-related microbial functions [26,32]. Dan et al. reported on changes in neurotransmitter-related metabolic pathways, such as serotonin-, dopamine-, histidine-, and GABA-related metabolites, but these findings were exploratory and were based on some subgroup analyses [33]. Su et al. identified changes in microbial functional pathways such as decreased ubiquinol-7 biosynthesis and thiamine diphosphate biosynthesis that enhanced the machine learning classification of ASD but provided no pathophysiological mechanism or diagnostic validity for the disease [37].

In the overall picture, none of the metabolites or functional pathways were repeated in all of the studies included. The current data thus suggest the presence of heterogeneous metabolic changes in ASD and not a metabolic signature. The metabolites detected in these studies cannot be used as markers or mechanistic pathways until larger longitudinal studies can be done with standardized metabolomic methods and other potential confounders fully accounted for, including dietary factors, gastrointestinal status, medication exposure, and others.

Table 11 provides a summary of the currently available microbiome-targeted intervention studies and main methodological limitations. An open-label pilot study of 30 participants for 12 weeks evaluated the synbiotic SCM06 by Wong et al. [41]. Anxiety, sensory-related symptoms, abdominal pain, and some biological outcomes improved. These changes, however, are not statistically significant as they have not been confirmed by randomization and blinding nor by the inclusion of a control group and should be considered preliminary.

**Table 11:** Microbiome-targeted intervention studies in ASD ASD: autism spectrum disorder; GI: gastrointestinal; ADOS: Autism Diagnostic Observation Schedule

Study	Design	Intervention	Sample/duration	Main findings	Key limitation	Interpretation
Wong et al., 2026 [41]	Open-label, uncontrolled pilot	SCM06 synbiotic	30 ASD; 12 weeks	Reported improvements in anxiety, sensory symptoms, and abdominal pain	Small sample; no control or blinding	Preliminary evidence only
Kang et al., 2017 [40]	Open-label, uncontrolled study	Microbiota transfer therapy	18 ASD; 7-8 weeks plus follow-up	Reported reductions in GI symptoms and selected ASD-related outcomes	High risk of bias; no randomization, control, or blinding	Very low-certainty evidence
Santocchi et al., 2020 [39]	Double-blind randomized controlled trial	Probiotic supplementation	85 ASD; 6 months	No overall ADOS-Calibrated Severity Score benefit; selected subgroup improvements in GI, adaptive, and sensory outcomes	Subgroup analyses and attrition	No confirmed overall treatment effect

Kang et al. carried out a small, uncontrolled, open-label, microbiota transfer therapy study of 18 individuals [40]. Reductions in gastrointestinal symptoms and improvements in selected ASD-related outcomes were reported although the intervention included antibiotic pretreatment, bowel cleansing, and microbiota administration. This is because there was no randomization, comparator group, or blinding and the risk of bias for confounding, participant selection, and outcome measurements was high, which reduces the extent to which causes can be inferred. The results are therefore only hypothesis-generating, not proof of treatment efficacy.

Santocchi et al. investigated the effects of probiotic supplementation for six months in a double-blind, randomized, placebo-controlled study [39]. There was no overall significant difference in the primary ADOS-Calibrated Severity Score (CSS) outcome. Improvements were only reported in selected subgroups and secondary outcomes such as gastrointestinal symptoms, adaptive functioning, and sensory profiles. These findings in the subgroups should be viewed with caution due to the smaller sample sizes of the subgroups, multiple comparisons, and the potential for chance findings.

The evidence for interventions is overall very limited and mixed. There are no studies currently in existence that prove synbiotics, probiotics, and microbiota transfer therapy are beneficial for the treatment of routine gastrointestinal, sensory, and/or behavioral symptoms in those with ASD. Larger randomized controlled trials with prespecified primary endpoints and appropriate control groups, blinded assessment, standard gastrointestinal and sensory measurements, and sufficient follow-up are needed.

Discussion

Gastrointestinal symptoms are very common in ASD and often accompany sensory processing difficulties and behavioral problems. The prevalence of some type of gastrointestinal disturbance is as high as 90% in children with ASD and is tightly linked to sensory sensitivities, anxiety, and decreased adaptive functioning [7,19,43]. It is important to highlight the specific outcomes observed to date with probiotic and microbiota-targeted therapies in the discussion of intervention outcomes. The interventions had a significant effect on gastrointestinal discomfort, with functional abdominal pain dropping from 26.7% to 10% (p=0.001) [41]. The open-label microbiota transfer study was reported to have significant reductions in gastrointestinal symptoms and improvements in a number of behavioral outcomes. However, these results must be interpreted with great caution; the study sample may be small, there was no randomized control group, there was no blinding, and ROBINS-I V2 rated this study as having a high overall risk of bias. The level of risk for confounding, participant selection, and outcome measurement reduced the confidence that the observed changes were due to the intervention rather than other factors. Therefore, the results are preliminary in nature and should not be considered as proof of effectiveness [40]. The improvements in the ADOS-CSS scores, adaptive functioning, and sensory profile in the subgroup analyses suggest that targeted interventions could have meaningful effects on core and comorbid symptoms of ASD [39]. These data suggest the potential for microbiome-targeted interventions to be linked to some positive changes in gut-, sensory-, and behavior-related outcomes. However, there are only a few controlled trials, small samples of patients, subgroup results, and short follow-up periods, which makes it difficult to draw clear conclusions on the effectiveness of the therapy.

Sensory dysregulation is very common in someone with ASD and could be related to oral, tactile, auditory, vestibular, proprioceptive, and other sensory modalities. Previous studies found overall sensory modulation differences in children with ASD [44,45]. These studies are referenced for their historical and conceptual context, that is, for the purpose of providing historical and conceptual background, but not for their inclusion in the systematic evidence synthesis, which limits studies to those conducted between 2011 and 2026. Restricted eating patterns and less variety in diet, related to sensory differences, could lead to variation in gut microbiome composition. However, there is no evidence available to determine the temporal sequence of these relationships.

There is considerable measurement heterogeneity that limits the interpretation of sensory findings. Other studies employed study-specific symptom measures, dietary assessments, physician-administered interviews, and more general phenotypic evaluations, while some studies employed standardized measures, such as the Short Sensory Profile, Childhood Autism Rating Scale (CARS), ADOS, ADI subscales, modified Gastrointestinal Severity Index, and Autism Treatment Evaluation Checklist [20,26,30,38,39]. Sensory constructs also varied from oral sensitivity and sensory over-responsivity to hyperreactivity and hyporeactivity and disgust sensitivity and global sensory profiles. In addition, the majority of sensory findings were parent/caregiver reported rather than clinician rated or objective, such as fMRI. Thus, results obtained with the various instruments, respondents, and sensory domains should not be taken as measuring the same phenomenon.

Yap et al. conducted a shotgun metagenomic analysis of gut microbiome composition in 247 participants and noted minimal direct links between gut microbiome composition and diagnosis of ASD while noting that age, diet, and bowel consistency were more strongly associated with variation in gut microbiome [24]. From these cross-sectional results, the authors theorized that restricted interest with ASD and sensory preferences for food could lead to decreased dietary diversity, changes in stool consistency, and changes in the microbiome. This evidence provides a plausible diet-mediated pathway but not necessarily the sequence or causality of microbiome differences due to behavioral and dietary causes. Therefore, the association may be interpreted as being two-way, and the evidence available is not sufficient to conclude that microbial dysbiosis is the main cause of sensory dysregulation in ASD.

However, the results of microbiome assessments are not necessarily comparable, because they are done in different ways. Culture-based methods only detect organisms that will grow in the laboratory, and qPCR only detects organisms with known targets. While 16S rRNA sequencing can give a community-level description of the bacteria, results can differ depending on the DNA extraction method, the hypervariable region of the 16S gene that was amplified, the sequencing platform, the taxonomic database used, and the bioinformatic pipeline. The species and functional resolution of shotgun metagenomics is also limited by sequencing depth and the reference database. The studies of metabolomics evaluate biochemical products but do not directly examine the composition of microorganisms, while biopsy studies of the intestine examine mucosa-associated communities which can vary greatly from the fecal community.

Thus, the differences in taxa reported between studies, for example, in *Prevotella*, *Bifidobacterium*, *Lactobacillus*, *Faecalibacterium*, and the *Firmicutes*-to-*Bacteroidetes* ratio, could be due to analytical differences as well as real biological variation. The results should thus be viewed as general trends and patterns that are dependent on the method used and should not be interpreted as a general specific microbiome for ASD.

In alignment with this model, several studies have demonstrated alterations in microbial diversity, as well as variations in specific taxa levels, including *Bifidobacterium*, *Lactobacillus*, and *Faecalibacterium prausnitzii*, which are associated with the severity of gastrointestinal symptoms and sensory processing difficulties [10,28,35]. Some results indicate that the detected differences in microorganisms are related not to the diagnosis of ASD but rather to dietary restriction and sensory-related food preferences. It cannot be confirmed by the evidence, however, that this is the proposed pathway.

There is some evidence available to examine the potential for sensory-based interventions to support people with ASD, but the quality and quantity of the evidence are highly variable. The 2024 systematic review and meta-analysis study highlighted positive outcomes of sensory integration therapy on certain sensory, motor, behavioral, and functional outcomes, primarily in studies conducted with Korean children [46]. The results of this review are specific to the population and context investigated and should not be extrapolated to other children with ASD or other clinical and cultural contexts without further validation.

The effectiveness of aquatic therapy as a multisensory approach for children with ASD has also been investigated. Selected aspects of social competence and quality of life were found to be improved after aquatic therapy in a mixed-methods study [47]. It is important to note that these results are only valid for this study and may not reflect overall improvement in areas of physical and adaptive functioning.

Children with ASD have been reported to be more sensitive to the oral qualities of food and are less likely to eat it and more likely to refuse a wider variety of foods [22]. This association encourages continued research on sensory-informed feeding interventions. But the key studies used in the review did not yield enough controlled evidence to draw conclusions that sensory desensitization consistently lowers oral hypersensitivity, oral mouthing, and/or food selectivity or that it then affects the composition of gut microbiota.

Individualized sensory regulation supportive strategies include occupational therapy and environmental modifications. But there is a lack of uniformity in the evidence base for combined intervention models, and clear conclusions about their effectiveness will require good-quality primary research and professional consensus.

There is a variation in the methodology, study design, population, comparator, intervention protocol, outcome measurement, and methodological quality of the evidence of sensory-based interventions. Meanwhile, Camarata et al. offered a methodological analysis, emphasizing the challenge of separating intervention-specific sensory effects from concurrent behavioral support and other nonspecific therapeutic aspects [48]. It is a methodological source and not a primary source of clinical effectiveness. Before general therapeutic statements can be made, a controlled trial with a well-defined intervention, well-elaborated sensory outcomes, blinded assessment if possible, and adequate follow-up is required. Additional research is also needed to ascertain if long-term changes in the vestibular, proprioceptive, auditory, or oral sensory regulation are followed by measurable long-term changes in dietary patterns, gastrointestinal symptoms, or gut microbiota composition.

Although the gut-brain-microbiome loop is an intriguing concept, existing research is predominantly of a cross-sectional nature. To control for sensory profiles, dietary intake, and the severity of autistic traits, longitudinal and experimental designs are essential. Standardization of the intervention protocol, the sensory assessment tools, and the microbiome sequencing protocol is also vital.

The interconnections between sensory regulation, dietary restriction, gastrointestinal symptoms, and gut microbiota composition are not completely understood and may be interconnected in ASD. The results of Yap et al. indicate that dietary preferences and associated behavioral traits could contribute to the observed microbiome differences, though this is preliminary and based on cross-sectional data [24]. All interventions should therefore be considered as separate categories of interventions with varying levels of evidence: sensory integration therapy, aquatic therapy, feeding support, dietary management, and microbiome-targeted approach. None of these approaches has been shown to be consistently superior or to be effective for everyone with ASD in the published literature.

Limitations

This review is comprehensive; however, there are limitations to be acknowledged. Firstly, the methodological diversity of the studies included (study design, sample sizes, and methods of microbiome analysis) makes it difficult to draw direct comparisons and synthesize the results. Secondly, most of the studies were cross-sectional, precluding strong causal conclusions about the relationship of gastrointestinal dysfunction, microbiota imbalance, and sensory processing dysregulation in ASD. Thirdly, there are different outcome measures in the various studies, especially in sensory processing and gastrointestinal symptom assessment, that pose difficulties in developing a unified understanding of the nuanced interactions.

The majority of studies included had a cross-sectional design, while only a few studies had a controlled interventional or longitudinal design. The overall evidence therefore suggests that there are associations between gastrointestinal dysfunction, the composition of gut microbiota, dietary patterns, and sensory dysregulation. It does not indicate a temporal direction or provide evidence of the role of microbial dysbiosis in the initiation and/or exacerbation of sensory symptoms. The possible causal pathways mentioned herein should thus be considered as hypotheses awaiting testing in longitudinal studies and sufficiently sized randomized controlled trials.

Sensory outcome measurement was variable, with different constructs, instruments, reporters, and modalities used for measurement. There are a number of studies that used parent/carer-reported symptoms and some that used clinician-rated assessments, dietary proxies, or objective neuroimaging. This may result in limited cross-comparability and recall, observer, and measurement bias.

Heterogeneity was also present with regard to the assessment of the microbiome. The different types of culture-based testing, targeted qPCR, 16S rRNA sequencing, shotgun metagenomics, fungal profiling, metabolomics, phage sequencing, and biopsy-based analysis have varying degrees of detection, specimen source, taxonomic resolution, functional inferences, and bioinformatic processing. Data obtained using these methods can't be directly compared. Thus, the narrative synthesis detects general relationships or trends, but does not provide a unique microbial profile or biome for a specific organism or function in ASD.

Furthermore, the majority of studies in the review were conducted with small sample sizes; thus, findings are not easily generalizable. In addition, the brief duration of some intervention studies adds to concerns about the long-term applicability and sustainability of the benefits reported. Therefore, larger and more in-depth trials are required to validate the findings from smaller studies and to determine evidence-based clinical interventions.

Future Perspectives and Recommendations

Future research should explicitly articulate the main research goals and anticipated outcomes. To further elucidate the alterations in gut microbiota along with changes in behavior over time, long-term research to establish causality between gut microbiota dysbiosis and sensory dysfunction in ASD are required. Standardization of sensory processing and gastrointestinal symptom measures, together with the utilization of uniform methodologies for microbiota investigation, is essential for facilitating meaningful comparisons and replication across research. Further studies with larger and longer clinical trials are needed to assess the long-term effects of microbiome-targeted interventions, such as probiotics, synbiotics, and fecal microbiota transplantation, on gastrointestinal and sensory outcomes in ASD. Furthermore, some future research should investigate relationships between individual food components, gut microbiota composition, and sensory function to inform therapeutic dietary recommendations that might reduce gut and sensory issues. The implementation of tailored fiscal policies, training of medical doctors to be equipped to deliver patient-centered neurodiversity-affirming care, and coherent actions from healthcare professionals and caregivers are crucial to accommodate effective communication and respect the sensory needs and treat this population with dignity. This multidisciplinary approach is essential for proper management and to attain optimal clinical outcomes while providing continued support from childhood to adulthood.

## Conclusions

The association between gut microbiota composition, gastrointestinal symptoms, sensory dysregulation, and dietary patterns in ASD is an ongoing research interest. Despite the evidence of associations among these factors, the evidence available at present fail to prove that dysbiosis in the gut is the main mechanism for sensory symptoms. The relationship could go in both directions: changes in the microbiota can influence the gastrointestinal and sensory outcomes, and dietary exposure (food choice, dietary variety, eating behaviors) can influence microbiota changes. Thus, the observed differences in the microbiome might be downstream effects of an eating pattern that lacks sensory awareness and not a primary contributor to sensory dysregulation.

A few preliminary studies on probiotics, synbiotics, and fecal microbiota transplantation have shown improvement in certain gastrointestinal, sensory, and behavioral parameters. However, these findings are not considered very reliable due to methodological heterogeneity, a lack of large sample sizes, limited follow-up, strong cross-sectional evidence, and the possibility of biased intervention studies. Results from the open-label microbiota transfer study are purely exploratory, especially when viewed as evidence of efficacy.

Considering the diversity of ASD, an individualized and neurodiversity-informed management approach could include medical, nutritional, feeding, and sensory-based approaches as needed for each individual. To understand the sense or direction of the relationship, longitudinal studies should assess the microbiota, gastrointestinal status, exposure to medications and antibiotics, stool characteristics, food selectivity, and sensory profiles over time. However, before the use of microbiome-targeted interventions can be recommended for clinical practice, sufficiently powered randomized controlled trials must be conducted first.
